# Blood type susceptibility to SARS-CoV-2 at a tertiary hospital in Accra, Ghana

**DOI:** 10.1128/spectrum.01108-24

**Published:** 2025-03-25

**Authors:** Prince N. Odoom, Olayinka S. Okoh, Yaa Y. Asare, Clara O. Mac-Arthur, Judith D. Azumah, Albert Mensah, Akua K. Yalley, Kwamena W. Sagoe, Nicholas I. Nii-Trebi

**Affiliations:** 1 Department of Medical Microbiology, University of Ghana Medical School 63533 , Accra, Ghana; 2 Department of Chemical Sciences, Anchor University, Lagos, Nigeria; 3 Anchor University Centre for Global Health, Lagos, Nigeria; 4 Department of Biochemistry and Biotechnology, Kwame Nkrumah University of Science and Technology 98763 , Kumasi, Ghana; 5 Department of Medical Laboratory Sciences, School of Biomedical and Allied Health Sciences, University of Ghana, Accra, Ghana; 6 Holy Child Catholic Hospital, Sekondi-Takoradi, Ghana; University of North Dakota, Grand Forks, North Dakota, USA

**Keywords:** SARS-CoV-2, COVID-19 susceptibility, seroprevalence, blood donors, blood grouping

## Abstract

Pandemics from viral outbreaks, such as that caused by SARS-CoV-2, have significant impacts worldwide. The factors that underlie differential susceptibility to severe COVID-19 outcomes are not fully understood. The role of the ABO blood group in the outcome of SARS-CoV-2 infections remains to be clarified in different populations. This study described the SARS-CoV-2 seroprevalence and examined the association of the ABO blood group with COVID-19 disease among apparently healthy and COVID-19 patients at the Korle Bu Teaching Hospital, Accra. The study involved 277 participants comprising 200 healthy individuals and 77 PCR-confirmed COVID-19 patients with mild or severe symptoms. Anti-SARS-CoV-2 antibody assay (IgM/IgG) was performed, and ABO blood grouping was done on plasma samples using the reverse blood grouping method. Statistical analyses were performed in R for the association of socio-demographic parameters and ABO blood groupings of participants with SARS-CoV-2 infection status. The total SARS-CoV-2 seropositivity was 61.4% (157/277). Most of the participants (245/277, 88.4%) were unvaccinated. Of the 245 unvaccinated individuals, 127 (51.8%) were IgG reactive. A significant association was observed between ABO blood group and COVID-19 disease status. Antigen A participants had a higher probability of symptomatic infection than non-antigen A individuals. Blood group O appeared more protective than other blood types among the participants. Seropositivity was high among the participants studied—vaccinated and unvaccinated. Blood group A is associated with an increased risk of COVID-19, whereas blood group O appears protective. Further studies involving larger sample sizes are required to confirm these findings.

**IMPORTANCE:**

The transmissibility and virulence of SARS-CoV-2 and the severity of COVID-19 disease appeared to vary across nations and among populations. However, the factors that account for the differential susceptibility and COVID-19 outcomes are not well understood. The roles of host immune defense mechanisms and genetic makeup have been implicated. This study investigated the seroprevalence of anti-SARS-CoV-2 antibodies (IgM and IgG) in apparently healthy individuals and COVID-19 patients; using a reliable but inexpensive blood group typing based on direct hemagglutination technique and rigorous statistical analyses, we determined the association of ABO blood groups with COVID-19 disease. We found appreciably high seropositivity among the participants studied—both vaccinated and unvaccinated—and showed that blood type significantly influences SARS-CoV-2 infection and COVID-19 severity, with blood group A associated with severe COVID-19 disease, whereas blood group O appears protective. Further studies involving a larger sample size are required to confirm these findings.

## INTRODUCTION

Discovered in Wuhan, Hubei Province, China, in December 2019, the severe acute respiratory syndrome coronavirus 2 (SARS-CoV-2), the agent responsible for COVID-19 disease (1, 2), soon became a worldwide public health emergency (3, 4). Its rapid spread worldwide led to it being declared a pandemic by the World Health Organization (WHO) on 11 March 2020 (5). Within six months of its emergence, the virus spread across 233 countries in all six continents. Three years on, by May 2023, at least 689 million cases had been recorded globally, out of which 6,881,042 deaths occurred (6).

Of note, the transmissibility and virulence of SARS-CoV-2 and the severity of COVID-19 disease appear to vary across nations and among populations. However, the factors accounting for these differences, especially in regions like sub-Saharan Africa, remain to be clarified. Specifically, why some infected individuals remain asymptomatic or suffer mild disease, while others are very vulnerable and suffer severe illness or even death, and why some exposed or vaccinated individuals develop protective antibodies and some do not, among others, are events that have not been well understood (7, 8). Host immune defense mechanisms and genetic makeup have been implicated to significantly influence the vulnerability to viral infections and the resulting disease (9, 10). Notably, previous studies have shown the ABO and Rh Blood group antigens to play a critical role in susceptibility and severity of viral diseases such as MERS-CoV, SARS-CoV, and HBV (11). Invariably, the ABO blood group antigens are crucial for determining an individual’s vulnerability to infectious microorganisms and the disease severity (12, 13).

A genome-wide association analysis on severe COVID-19 patients with acute distress respiratory syndrome (ADRS) (14) showed that loci 3p21.31 and 9q34.2 on chromosomes 3 and 9, respectively, have cross-replicating relationships; and the ABO blood type locus shares the same connection signal as locus 9q34.2. Studies done in the USA and China showed blood group A to be associated with more positive cases and greater COVID-19 severity. In contrast, people with blood group O had lower susceptibility and severity of COVID-19 disease (15, 16). However, there have been contradicting reports on the influence of ABO blood groups concerning vulnerability to SARS-CoV-2 and clinical outcomes of COVID-19 disease (17, 18). Since different ethnic and geographical locations have different immunogenetic characteristics, and immune responses to infections vary between individuals and various populations (19), the ABO blood group association with COVID-19 deserves investigation in different populations, including Ghana.

A valid, reliable, inexpensive blood grouping technique is a reverse typing approach based on direct hemagglutination—an approach that involves using confirmed A and B red cells to determine the ABO blood type based on the existence or lack of anti-A and anti-B antibodies in serum or plasma. This reverse typing is against the background that agglutination occurs when blood-type antigens found on erythrocytes are made to react with corresponding antibodies in plasma or serum. This study investigated the serological prevalence of anti-SARS-CoV-2 antibodies (IgM and IgG) in apparently healthy individuals and COVID-19 patients and determined the association of ABO blood type with the status and outcome of infection with SARS-CoV-2 at a tertiary hospital in Accra, Ghana.

## RESULTS

### Sociodemographic characteristics of study participants

Two hundred (200) apparently healthy individuals and 77 PCR-confirmed, symptomatic COVID-19 patients were studied. The ages of the participants ranged from 18 to 83 years. The mean age is 37 years, and the median age is 35 years. The participants were predominantly males (59.2%). The majority (53.3%) were married. For occupation, ‘Professional’ describes government workers and people in the private sector with a specific profession, including healthcare workers (HCWs), civil servants, engineers, and teachers. “Artisan” describes skilled workers who create objects or perform specific skills with their hands, including seamstresses, hairdressers, barbers, and drivers. The majority (43.0%) of the participants were professionals. Table 1 describes in detail the demographic characteristics of the study participants.

**TABLE 1:** Demographic characteristics of study participants (*N* = 277)^*a*^

Demographic characteristics	*N* (%)	Median (IQR)
Age groups		35 (18–83)
<20 years	3 (1.1)	
20–29 years	75 (27.1)	
30–39 years	95 (34.3)	
40–49 years	59 (21.3)	
50–59 years	26 (9.4)	
60–69 years	13 (4.6)	
70 + years	6 (2.2)	
Gender		
Male	164 (59.2)	
Female	113 (40.8)	
Marital status		
Single	78 (28.2)	
Married	190 (68.6)	
Widow(er)	9 (3.2)	
Educational qualification		
No School	9 (3.2)	
Primary	48 (17.3)	
Secondary/technical/vocational	115 (41.5)	
Tertiary	105 (38.0)	
Occupation		
Unemployed	31 (11.2)	
Professional	119 (43.0)	
Artisan	54 (19.5)	
Trader	73 (26.3)	

^*a*^ n—number of participants; N—total number of participants; and IQR—interquartile range.

**Fig 1:**
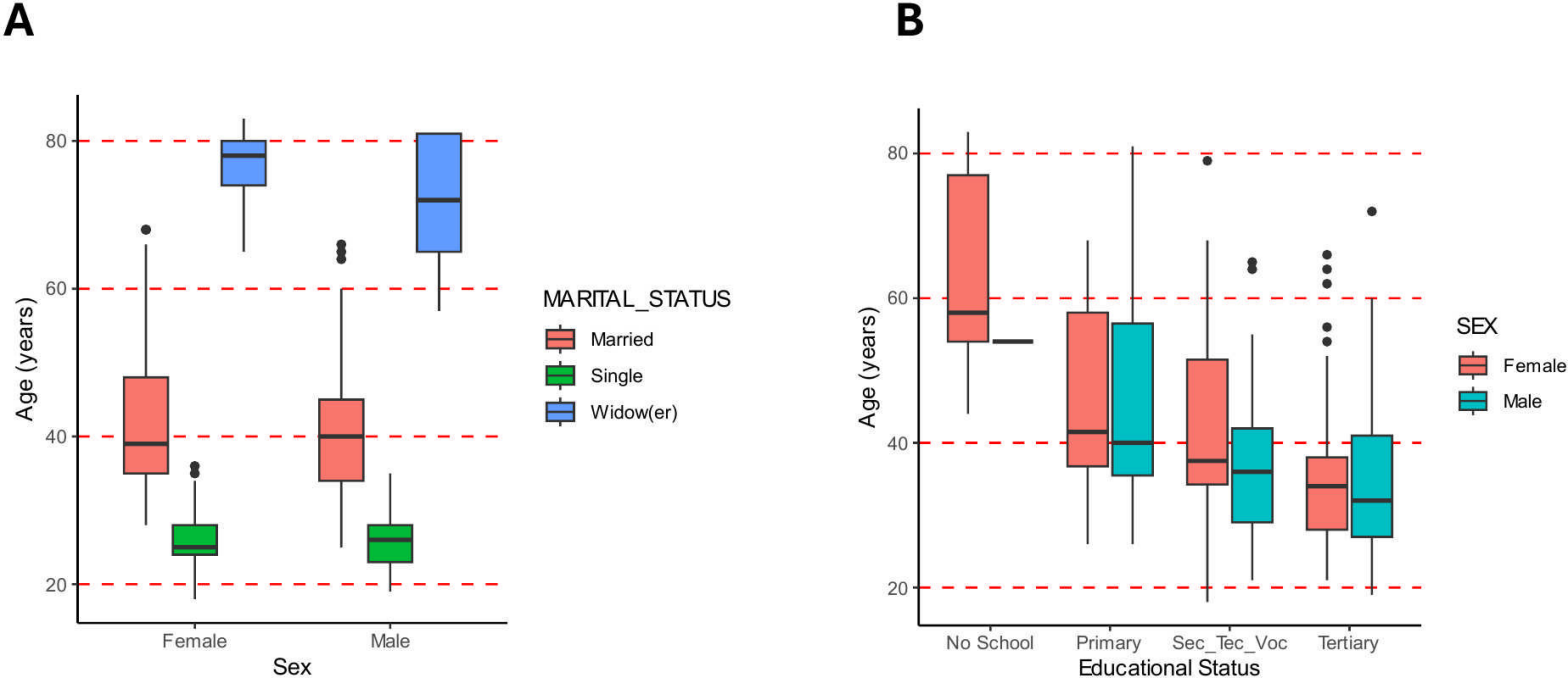
Sociodemographic characteristics of participants. (A) A boxplot showing the age ranges and marital status of male and female participants in the study. The figure shows that participants who were single were under 30 years, those married were within 30–50 years, and widows/widowers were above 60 years. (B) A boxplot showing the age ranges and educational levels of male and female participants of the study. The figure shows that for each educational level, the median age of female participants is higher than that of the male. It also shows that the higher the educational level, the lower the age of participants in the study.

Using the chi-square test, there was no significant association between all the variables assessed, as the *P*-values for each of the analyses were greater than 0.05, which is the level of significance. Further, even though no statistically significant association was found for any of the parameters tested, post-hoc analysis was conducted for confirmation, and the result of the pairwise comparison still showed that there was no significant association between all the variables assessed. Figure 1 presents details of the sociodemographic characteristics of the participants.

### COVID-19/SARS-CoV-2 infection and vaccination characteristics

Of the 200 apparently healthy participants, 3 (3/200, 1.5%) indicated suspicion of SARS-CoV-2 infection, given the COVID-19-related symptoms they experienced. None of the 200 had a laboratory test for the virus, and none indicated having had COVID-19 disease. Most of the participants (245/277; 88.4%) had not been vaccinated. Most (25/32) of those vaccinated belonged to the COVID-19 patients’ group.

### Serological prevalence of anti-SARS-CoV-2 among study participants

One hundred and fifty-seven out of the number studied (157/277, 56.7%) reacted positively to SARS-CoV-2 antibodies. In all, IgG seroprevalence was 53.4%. Antibody reactivity was recorded for IgG exclusively (*n* = 120), IgM exclusively (*n* = 9), and dual IgG/IgM (*n* = 28). Further details have been shown in Table 2A through C. Among the 200 healthy blood donors (Table 2B), the total seropositivity was 52.5% (105/200), with IgG seroprevalence being 49.5%. Table 2C shows the antibody reactivities among the 77 COVID-19 patients. The total seropositivity was 67.5% (52/77); the IgG seroprevalence was 63.6% (36/77).

**TABLE 2:** Seropositivity of study participants^*a*^^,^^*b*^

		*N* = 277		
A)		IgM		Total
		Non-reactive	Reactive	
IgG	Non-reactive	120	9	129
	Reactive	120	28	148
	Total	240	37	277

		*N* = 200		
B)		IgM		
		Non-reactive	Reactive	Total
IgG	Non-reactive	95	6	101
	Reactive	84	15	99
	Total	179	21	200

		*N* = 77		
C)		IgM		
		Non-reactive	Reactive	Total
IgG	Non-reactive	25	3	28
	Reactive	36	13	49
	Total	61	16	77

^*a*^ The table shows the seropositivity of various categories of study participants: respectively, panels A–C show the reactivities among (a) the entire study population of 277 participants, (b) shows that among the 200 apparently healthy individuals, and (c) shows the immunoglobulin (IgG/IgM) reactivity among the 77 COVID-19 patients. IgG reactivity appears higher in the COVID-19 group than in the entire or the apparently healthy groups.

^*b*^ IgG—Immunoglobulin G and IgM—Immunoglobulin M.

### ABO blood group association with SARS-CoV-2 exposure and COVID-19 disease outcomes

**Fig 2:**
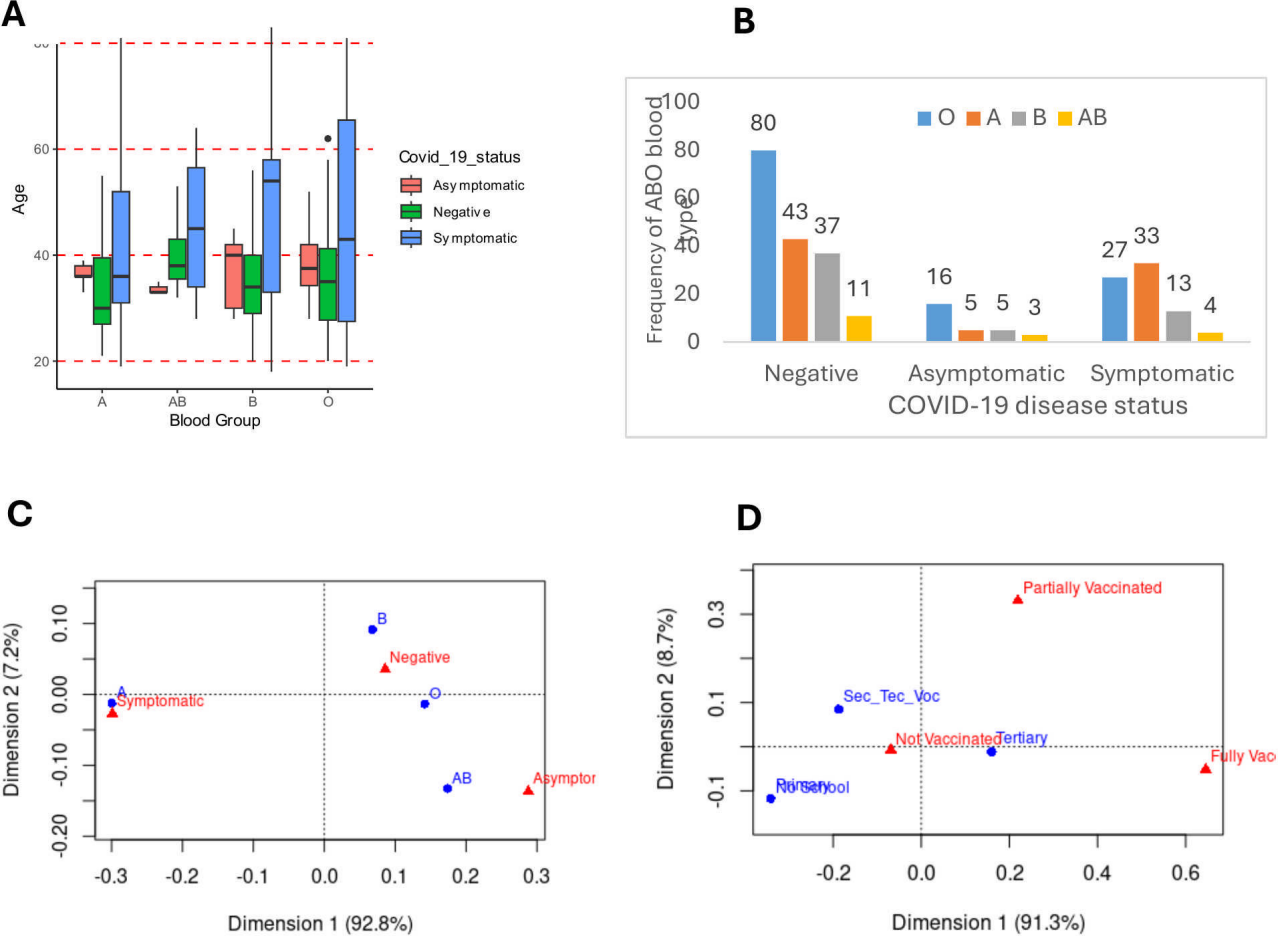
COVID-19 disease status across the ages (A), among the blood groups (B), and related association analysis (C and D). (A) shows ages 40 years and above associated more frequently with symptomatic disease, while asymptomatic and/or negative COVID-19 status usually occurred among individuals aged 40 years or below. (B) shows blood group “O” individuals most commonly present with a lack of COVID-19 disease status, while blood group “A” individuals are more prone to symptomatic COVID-19 disease. (C and D) display the correspondence analysis used to visualize relationships between blood group and COVID-19 disease status (C) and between educational level and vaccination status (Fig. 2D). (C) shows blood group A is strongly associated with symptomatic COVID-19 disease (*P* = 0.018); blood group AB is closely associated with asymptomatic status; and blood group O is strongly associated with negative COVID-19 status. (D) shows that the higher one’s level of education, the more willing or higher the chances of getting fully vaccinated.

Most (123/277, 44.4%) of the study participants were of blood group O. The other blood groups recorded are A (29%), B (20%), and AB (7%) in order of decreasing frequency (Fig. 2A). Logistic regression analyses were performed to predict a categorical outcome with multiple predictors, including age group, marital status, occupation, sex, educational level, and vaccination status in addition to the blood group in relation to the dependent variable—COVID-19 disease status (symptomatic or asymptomatic).

Regarding the risk of SARS-CoV-2 infection, the logistic regression results showed that occupation, specifically being either a student or trader, constitutes significant risks, with *P*-values of 0.0197 and 0.0274, respectively. Furthermore, low (pre-tertiary) educational level, namely primary and secondary/technical/vocational, increases the risk of SARS-CoV-2 infection, as indicated by *P*-values of 0.0450 and 0.0311, respectively. In terms of age, symptomatic disease occurred more frequently among individuals who were 40 years and above, while asymptomatic and/or negative COVID-19 status usually occurred among individuals who were 40 years or below (Fig. 2A).

Further, with respect to blood group predisposition, the results indicated that individuals with blood group O are less susceptible to SARS-CoV-2 and severe COVID-19 disease than blood group A individuals who are more susceptible (Fig. 2B). This is confirmed by the correspondence analysis (Fig. 2C), which shows that blood group A is strongly associated with symptomatic COVID-19, while blood group AB is closely associated with asymptomatic condition, whereas blood groups B and O, on the other hand, are closely associated with negative COVID-19 status.

While the correspondence analysis shows that the educational level is not strongly associated with any category of vaccination status (not vaccinated, partially vaccinated, or fully vaccinated), when considered at two levels—vaccinated and not vaccinated, the educational level was found to significantly influence vaccination (*P* = 0.001921). Specifically, the higher the educational level, the more willing one is to be vaccinated (Fig. 2D). The association of age group with vaccination status was underscored by the Cochran–Armitage test for trend, which showed that age group significantly (*P* = 0.006305) influences one’s willingness to be vaccinated.

### RT-PCR results from healthy blood donors

RT-PCR results of the 200 apparently healthy individuals (blood donors) indicated that 29 (14.5%) were SARS-CoV-2 positive. Of note, 16 (55.2%) of these 29 SARS-CoV-2 RT-PCR positive individuals were of blood group O; 5 (17.2%) were of blood groups A and B, and 3 (10.3%) were of blood group AB.

## DISCUSSION

The factors of spread and determinants of disease outcome following exposure to SARS-CoV-2 remain to be well understood in various populations. Male sex, comorbidities, smoking, and age have been described to constitute adverse prognostic factors for COVID-19 (20, 21). The role of host genetics remains to be well understood. This study determined the association of the ABO blood group, a host genetic marker, with susceptibility to SARS-CoV-2 infection and COVID-19 disease outcome. SARS-CoV-2 infection was defined by IgG/IgM seropositivity and viral nucleic acid detection in nasopharyngeal swabs by the RT-PCR technique.

The total seropositivity (IgG and IgM) recorded for the entire study population was 56.7%, and that for the blood donor sub-population was 49.5%. The IgG seroprevalence among the study population was 53.4%. This level appears higher than that obtained in an earlier study in Kumasi, the second largest city in Ghana, where a prevalence of 41.2% was recorded (22), as well as in other African countries. In Kenya, for example, a prevalence of 42.3% was reported among truck drivers (23); among the blood donors in the Democratic Republic of Congo, 31.4% seroprevalence was reported (24). In Nigeria, 42% was reported among blood donors (25); and in South Africa, a seroprevalence of 41% has been reported among COVID-19 patients in the public sector (26). Invariably, the seroprevalence reported in sub-Saharan Africa at the time of this study was below 50%. Various factors, including vaccination coverage, extent of exposure, and immune factors, might account for the low levels reported. Besides, not all individuals produce detectable antibodies or develop immunity after vaccination (27), in which case host genetics and viral characteristics could be implicated (28, 29).

Individuals with blood group O dominated among the study participants. This predominance of blood group O has been observed in various studies in populations of diverse ethnic backgrounds (30–32). Thus, by inference, these findings may suggest that blood group O is predominant in the Ghanaian population even though statistical analyses showed no significant association between blood group and susceptibility to SARS-CoV-2. Blood group O dominated among the negative control or SARS-CoV-2 uninfected individuals as well as among the asymptomatic patients. On the contrary, more blood group A individuals were among the symptomatic patients. Furthermore, a significant association was observed between blood group A and the development of symptomatic COVID-19 disease. These findings suggest that blood group O might confer some level of protection against the virus compared to the other blood groups. Blood group A may be associated with unfavorable COVID-19 outcomes.

This finding agrees with reports from other populations that associated blood group O with protection against SARS-CoV-2 while blood group A constituted a risk factor for adverse outcomes of SARS-CoV-2 infection. In a study conducted in Turkey, for example, blood group A individuals were found to be at increased risk of suffering from SARS-CoV-2 infection (OR = 1.45; 95% CI: 1.061–1.921) as compared to blood group O, which was associated with reduced risk to infection (OR = 0.686; 95% CI: 0522–0.903). In Nigeria, Kotila and colleagues (33) described blood groups B and AB as risk factors for SARS-CoV-2 infection, while blood group O was associated with protection against COVID-19. However, the mechanism for the protective effect of blood group O as against blood group A remains to be fully explained. Nevertheless, interference with binding between the viral S protein and cellular receptor ACE2 by anti-O agglutinins has been implicated [31] [32].

In summary, the seroprevalence of the studied population was high. This might suggest that many people were protected against SARS-CoV-2, whether antibody protection was gained either through vaccination or naturally through exposure. Blood group A appears to be a risk factor for developing severe COVID-19, while blood group O may provide some protection against SARS-CoV-2. Individuals with blood group A are prone to SARS-CoV-2 infection and are likely to have unfavorable COVID-19 outcomes. Accumulation of these data may enhance our understanding of differential immune responses and vulnerability to developing severe illness and inform preferential management strategies for COVID-19 patients.

## MATERIALS AND METHODS

### Study site and study design

The study employed both cross-sectional and experimental design. Healthy individuals were drawn from the blood bank, and COVID-19 patients were seen at the emergency unit, both at the Korle Bu Teaching Hospital (KBTH) in Accra, Ghana. Laboratory investigations were conducted at the Virology Laboratory, Department of Medical Microbiology, University of Ghana Medical School, and the National Public Health Reference Laboratory, also on the KBTH premises.

### Study population

Healthy individuals selected from among blood donors and PCR-confirmed COVID-19 symptomatic patients who were on admission at the Korle Bu Teaching Hospital Critical Decision-Taking Unit (CDTU) who were 18 years and above were eligible to participate in the study. Participants were selected randomly from those who satisfied the eligibility criteria and consented voluntarily by giving written informed consent. Eligibility criteria include willingness to undergo one blood draw irrespective of whether one has undergone vaccination. The total sample size for the study was 277, which comprised 200 apparently healthy individuals (blood donors) and 77 symptomatic PCR-confirmed COVID-19 patients.

### Sampling technique and data collection

A convenience sampling method was employed in this study. The study investigators did participant engagement and recruitment in collaboration with the Psychosocial Support Team responsible for COVID-19 counseling before testing. Every effort was made to enroll subjects across various ages to calculate and compare age-specific seroprevalence. The study’s objective was clearly explained to all participants for their understanding. All ethics procedures outlined in the ethics and safety considerations section of this proposal were followed to recruit consenting participants. A semi-structured questionnaire was administered to participants, who were interviewed to obtain their socio-demographic, clinical, and SARS-CoV-2 exposure, testing, and vaccination information. Subsequently, about 3 mL of venous blood was collected into EDTA anticoagulant tubes for serological testing of SARS-CoV-2 and blood grouping. Swabs from the respiratory mucosa of the oropharyngeal area were also collected from the 200 healthy (asymptomatic) blood donors and placed into a sterile transport tube containing a 2–3 mL viral transport medium (34) to be used for confirmation of SARS-CoV-2 infections. Blood samples and oropharyngeal swabs were appropriately transported with triple layer packaging at 4°C (ice chest with ice packs) to the Virology Unit, University of Ghana Medical School (UGMS) Microbiology Department and processed within 2 h of collection. Blood samples were centrifuged at 3000 rpm for 10 min to separate the plasma from the red blood cells. The plasma was preserved at −20°C for serological and blood grouping analysis.

### Anti-SARS-CoV-2 assay

The study employed a newly obtained COVID-19 IgG/IgM Rapid Test kit (Qingdao Hightop Biotech Company, Limited) to assess the serostatus of study participants. The COVID-19 IgG/IgM Rapid Test kit is a lateral flow immunochromatographic assay designed to test whole blood, serum, or plasma for COVID-19-specific IgG/IgM antibodies. The test was performed following the directions of the manufacturer. Briefly, 10 µL of plasma was added to the sample well in the test device. Two drops of sample buffer were added. The results were observed and recorded at room temperature within 15–20 min of incubation.

### SARS-CoV-2 viral RNA extraction and detection

SARS-CoV-2 viral RNA was extracted from the oropharyngeal swab samples using the Qiagen QIAamp viral RNA mini kit. The extractions were done following the kit instructions. Extracts were assayed for the presence of SARS-CoV-2 RNA by reverse-transcription qPCR using a Standard M nCoV Real-Time detection kit, according to the manufacturer’s instructions. This assay system is based on TaqMan probe real-time fluorescent RT-PCR technology, which uses probes and primers that target the ORF1ab (RdRp) gene and the E gene of the novel coronavirus (2019-nCoV) in the oropharyngeal swab, nasopharyngeal swab, and sputum specimens from humans.

### Reverse blood grouping

For blood grouping by the reverse typing approach, 3 mL of blood was drawn from two individuals with known blood groups—A and B. Blood was drawn into appropriately labeled EDTA tubes. The plasma was separated from the red blood cells through centrifugation for 10 min at 3,000 rpm. The red blood cells were washed three times with 5 mL of normal saline through centrifugation at 1,000 rpm for 3 min. A 5% suspension of the RBC sediment was prepared in normal saline (1 part of RBC added to 19 parts of normal saline) and used for the blood group typing of test samples, using the known blood group plasma as a test control.

A blood grouping test was done in a test tube. For each sample, two test tubes were labeled as A and B. Two drops of test plasma were added, after which one drop of the A and B cell suspensions was added to respective test tubes. The tubes were centrifuged at 1000 rpm for 1 min, after which the cells were thoroughly resuspended and macroscopically examined for agglutination. The absence of agglutination was confirmed by microscopic examination. The results were interpreted as shown in Table 3.

**TABLE 3:** Results interpretation chart for reverse blood grouping^*a*^^,^^*b*^

Unknown plasma with		Interpretation
Blood group A cells	Blood group B cells	Blood group
−	^+^	A
^+^	−	B
^+^	^+^	O
−	−	AB

^*a*^ + means agglutination.

^*b*^ − means no agglutination.

### Data management and statistical analysis

Data were entered in Microsoft Excel in 2019. Seroprevalence was estimated as the proportion of participants with a positive serological test result. A descriptive statistical analysis was performed. Categorical variables were expressed as percentages and continuous data as mean and standard deviation (mean ± SD). Statistical analyses were also carried out in detail in R version 4.4.2 (35) using RStudio (36), following standard analytical methods for disease association studies. Needed packages including readr (37), dplyr (38), ggplot2 (39), ggcorrplot (40), rcompanion (41), and DescTools (42) were loaded; and appropriate functions were called for the chi-square test of independence, logistic regression, correspondence analysis, and Cochran–Armitage trend test. The chi-square test of independence was used to test statistical significance between categorical variables. Logistic regression was to assess whether multiple variables (covariates) assessed during the studies significantly influenced the outcome of the COVID-19 infection or not. The correspondence analysis was used to assess the level of association between ABO blood groups and COVID-19 status, while the Cochran–Armitage trend test was used to assess whether an ordinal predictor like educational level influences COVID-19 status and whether the educational level also influences COVID-19 vaccination. The level of acceptable statistical significance was set at *P* < 0.05.

## References

[1] Khan S, Nabi G, Han G, Siddique R, Lian S, Shi H, Bashir N, Ali A, Shereen MA. 2020. Novel coronavirus: how things are in Wuhan. Clin Microbiol Infect 26:399–400. doi:10.1016/j.cmi.2020.02.005 32058086 PMC7129990

[2] Shereen MA, Khan S, Kazmi A, Bashir N, Siddique R. 2020. COVID-19 infection: origin, transmission, and characteristics of human coronaviruses. J Adv Res 24:91–98. doi:10.1016/j.jare.2020.03.005 32257431 PMC7113610

[3] Eurosurveillance editorial team. 2020. Note from the editors: World Health Organization declares novel coronavirus (2019-nCoV) sixth public health emergency of international concern. Euro Surveill 25:200131e. doi:10.2807/1560-7917.ES.2020.25.5.200131e PMC7014669 32019636

[4] Wu F, Zhao S, Yu B, Chen YM, Wang W, Song ZG, Hu Y, Tao ZW, Tian JH, Pei YY, Yuan ML, Zhang YL, Dai FH, Liu Y, Wang QM, Zheng JJ, Xu L, Holmes EC, Zhang YZ. 2020. A new coronavirus associated with human respiratory disease in China. Nature 579:265–269. doi:10.1038/s41586-020-2008-3 32015508 PMC7094943

[5] Hu B, Guo H, Zhou P, Shi ZL. 2021. Characteristics of SARS-CoV-2 and COVID-19. Nat Rev Microbiol 19:141–154. doi:10.1038/s41579-020-00459-7 33024307 PMC7537588

[6] Worldometer. 2023. COVID-19 coronavirus pandemic. Available from: https://www.worldometers.info/coronavirus/#countries

[7] Zhang JJ, Dong X, Liu GH, Gao YD. 2023. Risk and protective factors for COVID-19 morbidity, severity, and mortality. Clin Rev Allergy Immunol 64:90–107. doi:10.1007/s12016-022-08921-5 35044620 PMC8767775

[8] Stouten V, Hubin P, Haarhuis F, van Loenhout JAF, Billuart M, Brondeel R, Braeye T, Van Oyen H, Wyndham-Thomas C, Catteau L. 2022. Incidence and risk factors of COVID-19 vaccine breakthrough infections: a prospective cohort study in Belgium. Viruses 14:802. doi:10.3390/v14040802 35458532 PMC9029338

[9] Smatti MK, Alkhatib HA, Al Thani AA, Yassine HM. 2022. Will host genetics affect the response to SARS-CoV-2 vaccines? Historical precedents. Front Med (Lausanne) 9:802312. doi:10.3389/fmed.2022.802312 35360730 PMC8962369

[10] Carsetti R, Zaffina S, Piano Mortari E, Terreri S, Corrente F, Capponi C, Palomba P, Mirabella M, Cascioli S, Palange P, et al.. 2020. Different innate and adaptive immune responses to SARS-CoV-2 infection of asymptomatic, mild, and severe cases. Front Immunol 11:610300. doi:10.3389/fimmu.2020.610300 33391280 PMC7772470

[11] Mahmud R, Rassel MA, Monayem FB, Sayeed SKJB, Islam MS, Islam MM, Yusuf MA, Rahman S, Islam KMN, Mahmud I, Hossain MZ, Chowdhury AH, Kabir AKMH, Ahmed KGU, Rahman MM. 2021. Association of ABO blood groups with presentation and outcomes of confirmed SARS CoV-2 infection: a prospective study in the largest COVID-19 dedicated hospital in Bangladesh. PLoS One 16:e0249252. doi:10.1371/journal.pone.0249252 33826648 PMC8026078

[12] Harris M, Hart J, Bhattacharya O, Russell FM. 2023. Risk factors for SARS-CoV-2 infection during the early stages of the COVID-19 pandemic: a systematic literature review. Front Public Health 11:1178167. doi:10.3389/fpubh.2023.1178167 37583888 PMC10424847

[13] Torres-Alarcón CG, García-Ruíz A, Cañete-Ibáñez CR, Morales-Pogoda II, Muñoz-Arce CM, Cid-Domínguez BE, Montalvo-Bárcenas M, Torre GM la, Sandoval-López C, Gaytán-Guzmán E, Correo-Zamora JD. 2021. Blood system ABO antigens as risk factor for severity of SARS-CoV-2 infection. Gac Med Mex 157:174–180. doi:10.24875/GMM.20000498 34270530

[14] Ellinghaus D, Degenhardt F, Bujanda L, Buti M, Albillos A, Invernizzi P, Fernández J, Prati D, Baselli G, Asselta R, et al.. 2020. Genomewide association study of severe Covid-19 with respiratory failure. N Engl J Med 383:1522–1534. doi:10.1056/NEJMoa2020283 32558485 PMC7315890

[15] Zhao Y, Zhao Z, Wang Y, Zhou Y, Ma Y, Zuo W. 2020. Single-cell RNA expression profiling of ACE2, the receptor of SARS-CoV-2. Am J Respir Crit Care Med 202:756–759. doi:10.1164/rccm.202001-0179LE 32663409 PMC7462411

[16] Zietz M, Zucker J, Tatonetti NP. 2020. Testing the association between blood type and COVID-19 infection, intubation, and death. medRxiv:2020.04.08.20058073. doi:10.1101/2020.04.08.20058073 PMC7666188 33188185

[17] Pereira E, Felipe S, de Freitas R, Araújo V, Soares P, Ribeiro J, Henrique Dos Santos L, Alves JO, Canabrava N, van Tilburg M, Guedes MI, Ceccatto V. 2022. ABO blood group and link to COVID-19: a comprehensive review of the reported associations and their possible underlying mechanisms. Microb Pathog 169:105658. doi:10.1016/j.micpath.2022.105658 35764188 PMC9233352

[18] Rana R, Ranjan V, Kumar N. 2021. Association of ABO and Rh blood group in susceptibility, severity, and mortality of coronavirus disease 2019: a hospital-based study from Delhi, India. Front Cell Infect Microbiol 11:767771. doi:10.3389/fcimb.2021.767771 34796130 PMC8593001

[19] Brodin P, Davis MM. 2017. Human immune system variation. Nat Rev Immunol 17:21–29. doi:10.1038/nri.2016.125 27916977 PMC5328245

[20] Amoroso A, Magistroni P, Vespasiano F, Bella A, Bellino S, Puoti F, Alizzi S, Vaisitti T, Boros S, Grossi PA, Trapani S, Lombardini L, Pezzotti P, Deaglio S, Brusaferro S, Cardillo M, on behalf of the Italian Network of Regional Transplant Coordinating Centers. 2021. HLA and AB0 polymorphisms may influence SARS-CoV-2 infection and COVID-19 severity. Transplantation 105:193–200. doi:10.1097/TP.0000000000003507 33141807

[21] Ben Shachar S, Barda N, Manor S, Israeli S, Dagan N, Carmi S, Balicer R, Zisser B, Louzoun Y. 2021. MHC haplotyping of SARS-CoV-2 patients: HLA subtypes are not associated with the presence and severity of COVID-19 in the Israeli population. J Clin Immunol 41:1154–1161. doi:10.1007/s10875-021-01071-x 34050837 PMC8164405

[22] Struck NS, Lorenz E, Deschermeier C, Eibach D, Kettenbeil J, Loag W, Brieger SA, Ginsbach AM, Obirikorang C, Maiga-Ascofare O, et al.. 2022. High seroprevalence of SARS-CoV-2 in Burkina-Faso, Ghana and Madagascar in 2021: a population-based study. BMC Public Health 22:1676. doi:10.1186/s12889-022-13918-y 36064368 PMC9441841

[23] Uyoga S, Adetifa IMO, Karanja HK, Nyagwange J, Tuju J, Wanjiku P, Aman R, Mwangangi M, Amoth P, Kasera K, et al.. 2021. Seroprevalence of anti-SARS-CoV-2 IgG antibodies in Kenyan blood donors. Science 371:79–82. doi:10.1126/science.abe1916 33177105 PMC7877494

[24] Mokono SO, Nanitelamio EPCL, Mbani CJ, Tabapika A, Taty T, Ontsira NENN, Moukassa D. 2022. Seroprevalence of SARS-CoV-2 among blood donors in the Republic of Congo. Open J Blood Dis 12:124–132. doi:10.4236/ojbd.2022.124013

[25] Olayanju O, Bamidele O, Edem F, Eseile B, Amoo A, Nwaokenye J, Udeh C, Oluwole G, Odok G, Awah N. 2021. SARS-CoV-2 seropositivity in asymptomatic frontline health workers in Ibadan, Nigeria. Am J Trop Med Hyg 104:91–94. doi:10.4269/ajtmh.20-1235 33185181 PMC7790104

[26] Hussey H, Vreede H, Davies M-A, Heekes A, Kalk E, Hardie D, van Zyl G, Naidoo M, Morden E, Bam J-L, Zinyakatira N, Centner CM, Maritz J, Opie J, Chapanduka Z, Mahomed H, Smith M, Cois A, Pienaar D, Redd AD, Preiser W, Wilkinson R, Chetty K, Boulle A, Hsiao N-Y. 2022. Epidemiology and outcomes of SARS-CoV-2 infection associated with anti-nucleocapsid seropositivity in Cape Town, South Africa. medRxiv:2022.12.01.22282927. doi:10.1101/2022.12.01.22282927

[27] Post N, Eddy D, Huntley C, van Schalkwyk MCI, Shrotri M, Leeman D, Rigby S, Williams SV, Bermingham WH, Kellam P, Maher J, Shields AM, Amirthalingam G, Peacock SJ, Ismail SA. 2020. Antibody response to SARS-CoV-2 infection in humans: a systematic review. PLoS One 15:e0244126. doi:10.1371/journal.pone.0244126 33382764 PMC7775097

[28] Sanchez-Mazas A. 2020. A review of HLA allele and SNP associations with highly prevalent infectious diseases in human populations. Swiss Med Wkly 150:w20214. doi:10.4414/smw.2020.20214 32297957

[29] Liu N, Zhang T, Ma L, Zhang H, Wang H, Wei W, Pei H, Li H. 2021. The impact of ABO blood group on COVID-19 infection risk and mortality: a systematic review and meta-analysis. Blood Rev 48:100785. doi:10.1016/j.blre.2020.100785 33309392 PMC7834371

[30] Bullerdiek J, Reisinger E, Rommel B, Dotzauer A. 2022. ABO blood groups and the risk of SARS-CoV-2 infection. Protoplasma 259:1381–1395. doi:10.1007/s00709-022-01754-1 35364749 PMC8973646

[31] Doku GN, Agbozo WK, Annor RA, Kisseh GD, Owusu MA. 2019. Frequency of ABO/Rhesus (D) blood groupings and ethnic distribution in the Greater-Accra region of Ghana, towards effective blood bank inventory. Int J Immunogenet 46:67–73. doi:10.1111/iji.12412 30604500

[32] Doku GN, Agbozo WK, Annor RA, Mawudzro PE, Agbeli EE. 2022. Frequencies and ethnic distribution of ABO and RhD blood groups in the Volta region of Ghana, towards effective blood bank services. Afr Health Sci 22:641–647. doi:10.4314/ahs.v22i1.74 PMC9382516 36032446

[33] Kotila TR, Alonge TO, Fowotade A, Famuyiwa OI, Akinbile AS. 2021. Association of the ABO blood group with SARS-CoV-2 infection in a community with low infection rate. Vox Sang 116:910–915. doi:10.1111/vox.13077 33529391 PMC8014178

[34] Prevention CfDCa. 2020. Lab advisory: updated interim guidelines for collecting, handling, and testing clinical specimens from persons under investigation (PUIs) for coronavirus disease 2019 (COVID-19). Available from: https://www.cdc.gov/locs/dynamic-temp/2020/updated_interim_pui_guidelines_for_covid-19.html

[35] Team RC. 2024. R: a language and environment for statistical computing. R Foundation for Statistical Computing, v4.4.2. Vienna, Austria. https://www.R-project.org.

[36] Team P. 2024. RStudio: integrated development environment for R, posit software. PBC, Boston, MA. http://www.posit.co.

[37] Wickham H, Hester J, Bryan J. 2024. readr: read rectangular text data, vR package version 2.1.5. https://CRAN.R-project.org/package=readr.

[38] Henry L, Müller K, Vaughan D. 2023. dplyr: a grammar of data manipulation, vR package version 1.1.4. https://CRAN.R-project.org/package=dplyr.

[39] Wickham H. 2016. ggplot2: elegant graphics for data analysis. Springer-Verlag, New York.

[40] Kassambara A. 2023. ggcorrplot: visualization of a correlation matrix using 'ggplot2', vR package version 0.1.4.1. https://CRAN.R-project.org/package=ggcorrplot.

[41] Mangiafico SS. 2024. rcompanion: functions to support extension education program evaluation., v2.4.36. Rutgers Cooperative Extension, New Brunswick, New Jersey. https://CRAN.R-project.org/package=rcompanion.

[42] Signorell A. 2025. DescTools: tools for descriptive statistics, vR package version 0.99.59. https://CRAN.R-project.org/package=DescTools.

